# *cyp51A* mutations, protein modeling, and efflux pump gene expression reveals multifactorial complexity towards understanding *Aspergillus* section *Nigri* azole resistance mechanism

**DOI:** 10.1038/s41598-024-55237-9

**Published:** 2024-03-14

**Authors:** Pooja Sen, Mukund Vijay, Himanshu Kamboj, Lovely Gupta, Jata Shankar, Pooja Vijayaraghavan

**Affiliations:** 1 Amity Institute of Biotechnology, Amity University Uttar Pradesh, Sector-125, Noida, Uttar Pradesh India; 2 Department of Biotechnology and Bioinformatics, Jaypee University of Information Technology, Solan, India

**Keywords:** Antimicrobials, Fungal pathogenesis, Protein structure predictions, Fungal infection

## Abstract

Black *Aspergillus* species are the most common etiological agents of otomycosis, and pulmonary aspergillosis. However, limited data is available on their antifungal susceptibility profiles and associated resistance mechanisms. Here, we determined the azole susceptibility profiles of black *Aspergillus* species isolated from the Indian environment and explored the potential resistance mechanisms through *cyp51A* gene sequencing, protein homology modeling, and expression analysis of selected genes *cyp51A*, *cyp51B*, *mdr1*, and *mfs* based on their role in imparting resistance against antifungal drugs*.* In this study, we have isolated a total of 161 black aspergilli isolates from 174 agricultural soil samples. Isolates had variable resistance towards medical azoles; approximately 11.80%, 3.10%, and 1.24% of isolates were resistant to itraconazole (ITC), posaconazole (POS), and voriconazole (VRC), respectively. Further, *cyp51A* sequence analysis showed that non-synonymous mutations were present in 20 azole-resistant *Aspergillus* section *Nigri* and 10 susceptible isolates. However, Cyp51A homology modeling indicated insignificant protein structural variations because of these mutations. Most of the isolates showed the overexpression of *mdr1*, and *mfs* genes. Hence, the study concluded that azole-resistance in section *Nigri* cannot be attributed exclusively to the *cyp51A* gene mutation or its overexpression. However, overexpression of *mdr1* and *mfs* genes may have a potential role in drug resistance.

## Introduction

*Aspergillus niger* and its related species are grouped in *Aspergillus* section *Nigri*, commonly known as black aspergilli. These species are unevenly distributed globally and are often isolated from clinical samples^1^. Black aspergilli infections are the third most common cause of *Aspergillus*-associated infections*,* leading to conditions such as otomycosis, onychomycosis, and pulmonary aspergillosis^2,3^.

Triazoles comprise the first-line treatment for aspergillosis however, long-term therapy and widespread use of azole-based pesticides in agriculture have raised concerns because of an increase in resistance to the medical triazoles in various *Aspergillus* species^4^. The acquired resistance is not only because of the fungicidal effect of azoles but also caused by azole exposure in clinical and environmental settings^5–8^. Multi-azole resistance has been reported in patients and from the environment across Europe, China Japan, The Middle East, and India^5,9–12^. Several authors have reported azole-resistant *Aspergillus* isolates, which correlates with the poor therapeutic outcome of azole, thereby limiting the treatment options^6,7,13,14^. Previous studies have reported that the antifungal drug susceptibility of itraconazole (ITC) against clinical and environmental isolates of *A. niger* and *A. tubingensis* showed higher minimum inhibitory concentration (MIC)^15–18^.

The azole drugs act via non-competitive binding to the Cyp51 enzyme, a sterol 14α-demethylase, of the ergosterol biosynthetic pathway. The azole inhibits ergosterol synthesis and disrupts cell membrane^19^. The mechanism of azole resistance in *Aspergillus fumigatus* has been extensively studied*.* Several mutations in the *cyp51A* gene and overexpression of this gene have been reported in azole-resistant *A. fumigatus* strains^20^. However, azole resistance mechanisms have not been extensively investigated in the *A. niger* complex. The molecular mechanism of azole resistance in section *Nigri* was first reported by Howard et al.^15^. Since then, a few reports have elaborated on the resistance mechanisms against *A. niger* complex via mutation analysis of the *cyp51A* gene or its expression. Pérez-Cantero et al.^21^ reported that *cyp51B* gene expression in the *Aspergillus* section *Nigri* was not inducible after azole exposure. However, the underlying azole resistance mechanisms in *Aspergillus* section *Nigri* have not been fully explored.

Identification of a relatively large number of clinical azole-resistant *A. fumigatus* isolates lacking the *cyp51A* mutations and comparative genomics studies in yeasts prompted the investigations on alternative mechanisms of azole resistance. This led to the discovery of the role of efflux pumps in azole resistance. Efflux pumps are categorized into two main classes: the major-facilitator superfamily (MFS) proteins, encoded by 278 genes, and ATP-binding cassette (ABC) proteins, encoded by 49 genes^22^. Tobin et al.^23^ identified two ABC transporter proteins; MDR1 and MDR2 in *A. fumigatus* based on cloning and sequence homology. Further, overexpression of MDR3 and MDR4 in ITC-resistant *A. fumigatus* strains was reported^24,25^. In a recent study, overexpression of efflux pump genes has been reported in azole resistant *A. niger* isolates^26^. Overexpression of efflux pump genes has also been observed in response to amphotericin B in *A. fumigatus.* This report suggested that the fungus adapts by overexpressing genes and proteins involved in drug efflux, representing a mechanism to develop resistance and survive against antifungal drugs^27^. Moreover, overexpression of efflux pumps genes like *abcC* in azole-resistant *A. fumigatus* isolates has been highlighted in other studies^28,29^.

Here, we aimed to determine the correlation between azole resistance and the *cyp51A* gene mutation in black *Aspergillus* isolates isolated from agricultural soil samples across India through antifungal susceptibility testing, gene sequencing, expression analysis of selected genes and protein homology modeling. Further, we attempted to elucidate the role of efflux transporter genes, *mdr1*, and *mfs*, in azole-resistant environmental isolates.

## Results

### Identification and antifungal susceptibility testing

**Figure 1:**
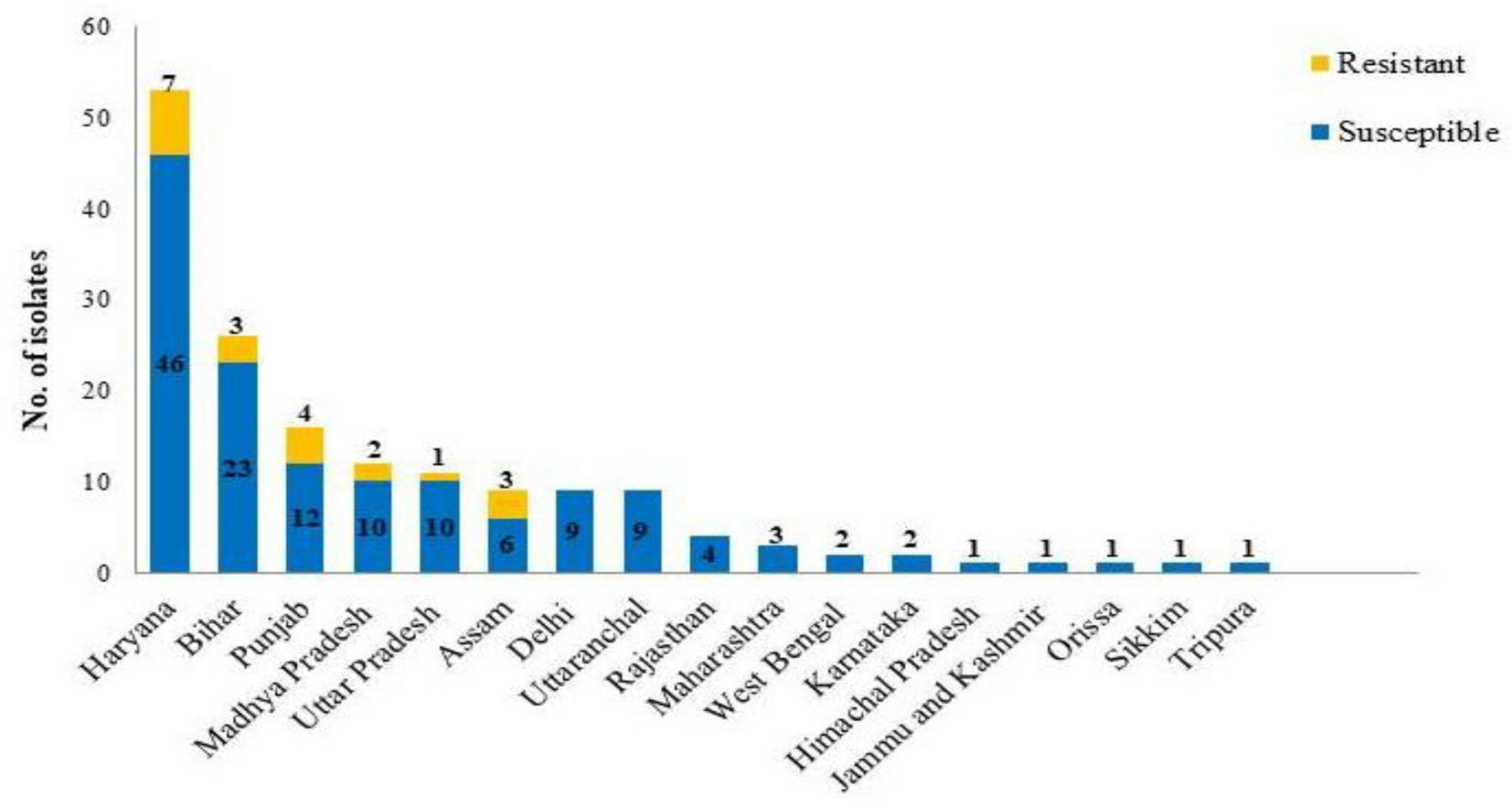
*Aspergillus* section *Nigri* isolates (n = 161) identified from soil samples collected from different states of India. The blue bars represent the number of susceptible isolates, and the yellow bars represent the number of azole-resistant *Aspergillus* section *Nigri* isolates.

**Figure 2:**
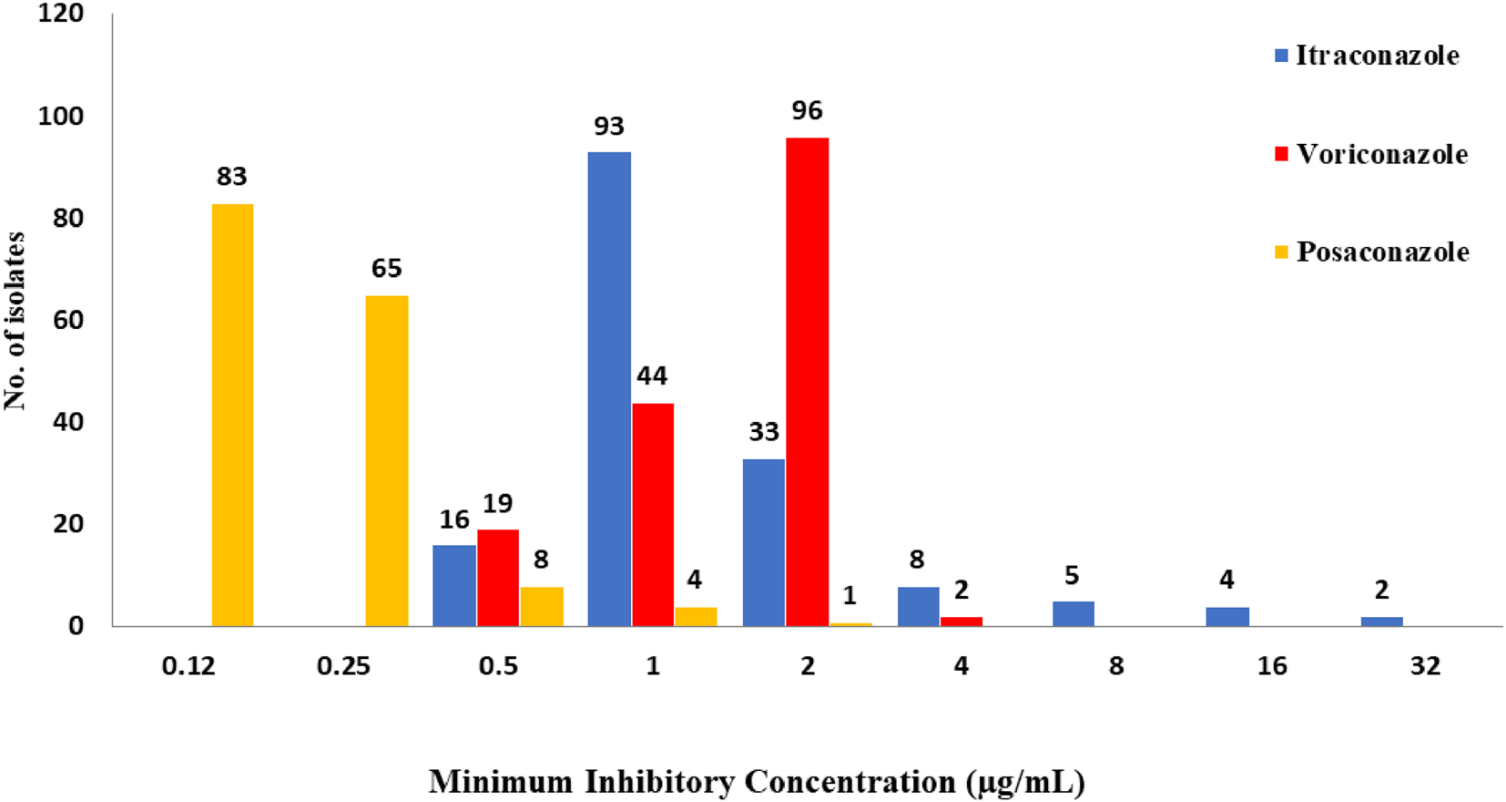
Distribution of *Aspergillus* section *Nigri* isolates based on the MIC values (n = 161). *Aspergillus* section *Nigri* was classified as resistant (R), when MIC was above the following ECV values: itraconazole ≤ 2 μg/mL, voriconazole ≤ 2 μg/mL, and posaconazole ≤ 0.5 μg/mL.

We screened 161 black aspergilli isolates isolated from 174 soil samples across India followed by antifungal susceptibility analysis against ITC, voriconazole (VRC), and posaconazole (POS). Results revealed that out of 161 isolates, 20 were resistance to at least one azole drug. Resistant isolates were obtained from the samples collected from Haryana (7/46), Bihar (3/23), Punjab (4/12), Madhya Pradesh (2/10), Uttar Pradesh (1/10 and Assam (3/6) region (Fig. 1). Furthermore, azole susceptibility revealed that 19/161 black aspergilli isolates was above its epidemiological cutoff value (ECV) of > 2 μg/mL for ITC. Five isolates had MIC > 0.5 μg/mL for POS (Table 1; Fig. 2). Four black aspergilli isolates were cross-resistant to ITC and POS. For VRC, all isolates (except AG1 and AU3) showed MICs below the ECV values (2 μg/mL), and only two isolates were cross-resistant to ITC and VRC (AU3 and AG1). We inferred that VRC and POS were the most effective triazoles against black aspergilli isolates, and ITC was the least effective triazole.

**Table 1:** *In-vitro* azole susceptibility profile of 161 *Aspergillus* section *Nigri* isolates.

Azole	MIC values (µg/mL)							
	0.12	0.25	0.5	1	2	4	8	> 8
Itraconazole	0	0	16	93	33	**8**	**5**	**6**
Voriconazole	0	0	19	44	96	**2**	0	0
Posaconazole	83	65	8	**4**	**1**	0	0	0

Number of isolates having MICs above ECVs (2 μg/mL for itraconazole and voriconazole; 0.5 μg/mL for posaconazole) are shown in bold.

### Molecular identification of black *Aspergillus* isolates

Molecular identification was performed for 20 azole-resistant black aspergilli isolates. Additionally, to provide a comparative context, we included 10 azole-susceptible black aspergilli isolates from the same geographical region as the resistant isolates. Using 18S internal transcribed spacer (ITS) and *β*-tubulin gene sequencing a total of 21 *A. niger* (13 resistant and 8 susceptible) and 9 *A. tubingensis* (7 resistant and 2 susceptible) isolates from the 30 *Aspergillus* section *Nigri* isolates were identified. All tested ITS and *β*-tubulin gene sequences displayed 99% to 100% nucleotide identity with the sequences available in NCBI database. The GenBank accession numbers generated for the submitted ITS sequences were OK342205, MZ305300, MZ305301, MZ305302 MZ305303, MZ292199, MZ292200, MZ292198, MZ292197, MZ292196, MW332573, MW282896, OQ938544, OQ938545, OQ938546, OQ935460, OQ935461, OQ935462, OQ935463, OQ935464, OQ935465, OQ935466, OQ935467, OR575635, OR575636, OR575637, OR575638, OR575639, OR575640 and for *β*-tubulin gene were OQ948174, OQ948175, OQ948176, OQ948177, OQ948178, OQ948179, OQ948180, OQ948181, OQ948182, OQ948183, OQ948184, OQ948185, OQ948186, OQ948187, OQ948188, OQ948189, OQ948189, OQ948190, OQ948191, OQ948192, OQ948193, OQ948194,OQ948195, OR584016, OR584017, OR584018, OR584019, OR584020, OR584021, OR584022, OR584023.

### Mutation analysis of the *cyp51A* gene

The *cyp51A* gene was sequenced and analyzed for all 20 resistant (13 *A. niger* and 7 *A. tubingensis*) and 10 susceptible (8 *A. niger* and 2 *A. tubingensis*) *Aspergillus* section *Nigri* isolates. The sequences of the *cyp51A* gene of *A. niger* and *A. tubingensis* resistant isolates were aligned with the wild-type strains NT_166526 and JF450924.1, respectively (Supplementary Figs. S1, S2). We identified 15 non-synonymous mutations among the *Aspergillus* section *Nigri* isolates. Table 2 shows the amino acid alterations in the *cyp51A* gene. Among these, several mutations were found in both susceptible and resistant isolates. In *A. niger*, the amino acid change Q228R was observed in both resistant and susceptible isolates. However, the mutation S346R in combination with Q228R was identified in various isolates, with different susceptibility to azole drugs. This included one azole-susceptible isolate (PU2), two isolates resistant to either ITC or POS (RK5 and MP6), two isolates displaying cross-resistance to ITC and VRC (AU3 and AG1), and two isolates with cross resistance to ITC and POS (AU4 and MP8). Further, the amino acid change V383L, R501Q, I244F, E254D, D253Y, T267Y, Y268H, E278Q and L303M were found only in three *A. niger* resistant isolates, that exhibited resistance to only ITC (MD2, PR4, PI5).In the case of *A. tubingensis,* amino acid substitution T321A in combination with V377I was observed in three ITC resistant isolates (FA9, FA4, and FA6). Additionally, one of the identified ITC resistant isolate (FA10) exhibited this substitution along with K477N mutation. Mutation T321A was also observed independently in ITC-resistant isolate (AH6), an isolate exhibiting cross resistant to both ITC and POS (PR2), and a susceptible isolate (AH1). Furthermore, a new amino acid substitution, V329I was detected in one ITC-resistant (PR9) and in one susceptible (UT1) *A. tubingensis* isolates.

**Table 2:** Amino acid substitutions observed in the *cyp51A* region of *Aspergillus* section *Nigri* isolates.

Species	Isolate code	MIC values (µg/mL)				Amino acid substitution
		S/R	ITC	POS	VRC	
*A. niger*	RK5	R	**16**	0.5	0.5	Q228R, S346R
	MD2	R	**4**	0.25	1	Q228R, V383L
	PI3	R	**4**	**1**	0.5	Q228R
	PR3	R	**4**	0.5	0.125	Q228R
	PR4	R	**4**	0.5	2	Q228R, I244F, E254D, D253Y, T267Y, Y268H, E278Q, L303M
	F1	R	**4**	0.25	1	Q228R
	PI5	R	**4**	0.25	–	Q228R, R501Q
	PU6	R	**4**	0.5	–	Q228R
	AU3	R	**8**	0.125	**4**	Q228R, S346R
	AU4	R	**16**	**1**	1	Q228R, S346R
	AG1	R	**4**	0.125	**4**	Q228R, S346R
	MP6	R	1	**2**	1	Q228R, S346R
	MP8	R	**8**	**1**	0.5	Q228R, S346R
	PI2	S	1	0.125	1	Q228R
	PI4	S	0.5	0.25	0.5	Q228R
	PU1	S	1	0.5	1	Q228R
	PU2	S	1	0.125	0.5	Q228R, S346R
	F2	S	1	0.5	2	Q228R
	TR1	S	1	0.125	1	Q228R
	PR5	S	1	0.125	0.5	Q228R
	J6	S	2	0.25	1	Q228R
*A. tubingensis*	FA9	R	**32**	− 0.125	− 1	V377I, T321A
	FA10	R	**16**	–0.125	− 2	K477N, T321A
	FA4	R	**8**	− 0.125	− 0.5	V377I, T321A
	FA6	R	**16**	0.5	2	V377I, T321A
	PR9	R	**8**	0.125	1	V329I, L492M
	PR2	R	**32**	**1**	0.5	T321A
	AH6	R	**8**	− 0.125	2	T321A
	UT2	S	2	0.125	2	V329I
	AH1	S	1	0.25	1	T321A

*S* Susceptible, *R* Resistant.

Bold letters depict MIC above ECV values: > 2 µg/mL for itraconazole and voriconazole and > 0.5 µg/mL for posaconazole.

### Cyp51A homology modeling and molecular docking

**Figure 3:**
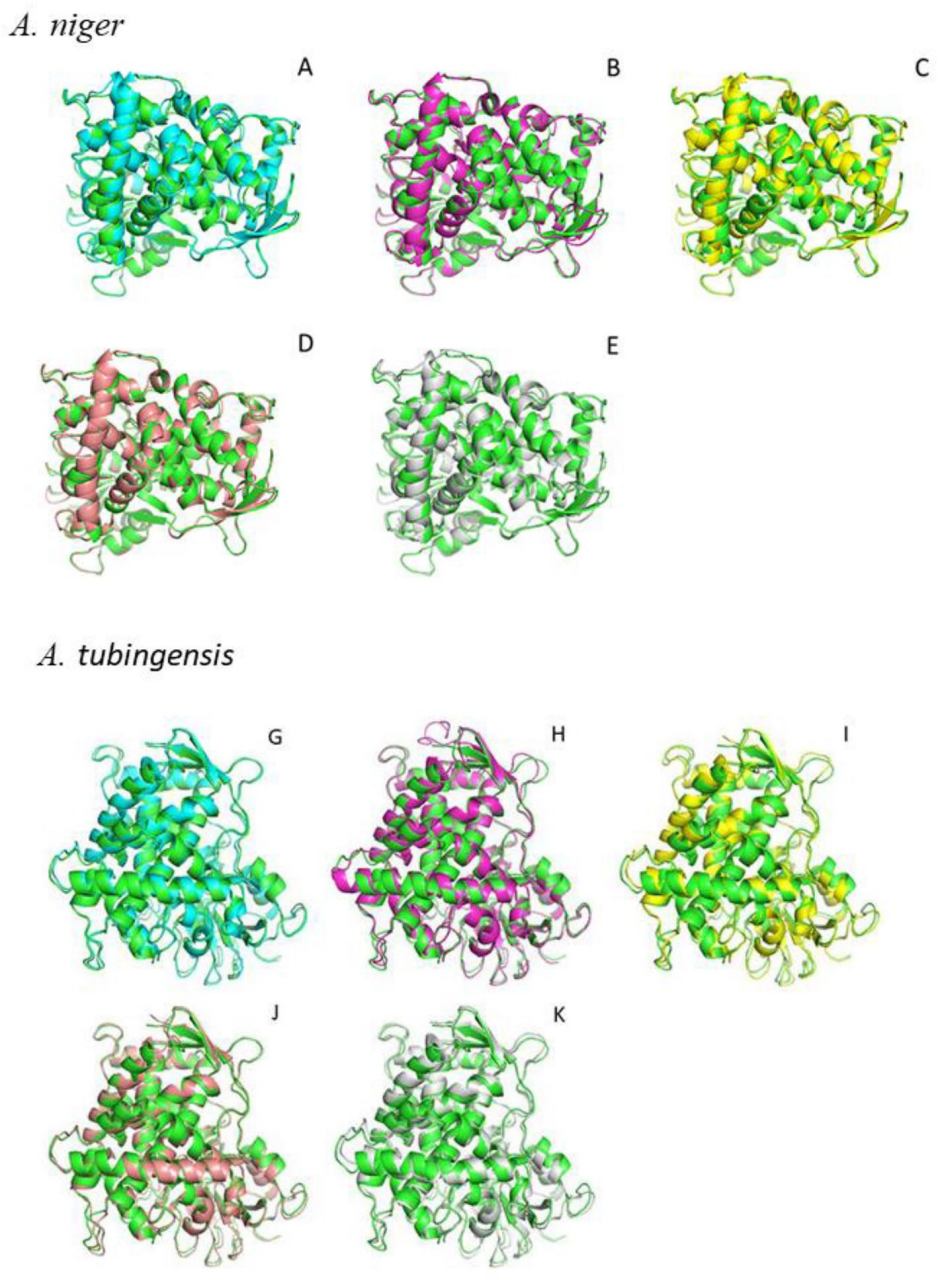
Ribbon representations of superimpositions of modeled (**A**–**E**) and (**G**–**K**) mutated Cyp51A protein structure with the wild-type protein of *A. niger* and *A. tubingensis* respectively. The green color structure represents the wild-type Cyp51A proteins. Models (**A**,**D**,**E**,**G**,**I**,**K**) correspond to structures associated with resistant isolates. Model (**J**) represents structure associated with susceptible isolate. In contrast, Models (**B**,**C**,**H**) represents structures associated with both susceptible and resistant isolates.

We constructed ten *Aspergillus* section *Nigri* Cyp51A homology models (A to L) to compare the amino acid profiles of azole-resistant isolates with those of wild-type isolates. Five mutation combinations observed in all *A. niger* isolates were: Q228R/V383L, Q228R/S346R, Q228R, Q228R/R501Q, and Q228R/I244F/D253Y/E254D/T267Y/Y268H/E278Q. Therefore, models A to E were constructed after incorporating these mutations in *A. niger* isolates (Fig. 3). Similarly, *A. tubingensis* models G to K were constructed for the mutations V329I/L492M, T321A, V377I/T321A, V329I, and K477N/T321A (Fig. 3), and two wild-type models F and L was constructed for *A. niger* and *A. tubingensis*, respectively. Table 3 showed the docking score of the different Cyp51A protein models of the aspergilli isolates with ITC and VRC. Compared to wild-type isolates, marked variations in the overall protein structure conferring resistance were not identified in models A to E, and G to K. The most negative docking score was obtained with model E for ITC, and docking scores were almost similar in all the models for VRC. H-bond interactions between the ligands (ITC and VRC) and Cyp51A protein structure were obtained in models A, D, H, J, K, and L. In model A, ITC interacted with CYS447, in model H, it interacted with TYR51, and in model K interaction was observed between ITC and TYR119, whereas, two H-bonds were formed between model J and ITC with TYR119 and SER360. VRC interacted with TYR119 via H-bond interactions in models A, H, I, J, K, and L, while, H-bond interactions were observed between CYS447 and VRC in models D and G.

**Table 3:** Docking scores of the different Cyp51A protein models of the aspergilli isolates with itraconazole and voriconazole.

Species	Model/Mutation	Ligand	Docking score (kcal/mol)
*A. niger*	A (Q228R/V383L)	Itraconazole	− 10.558
		Voriconazole	− 8.626
*A. niger*	B (Q228R/S346R)	Itraconazole	− 12.514
		Voriconazole	− 8.409
*A. niger*	C (Q228R)	Itraconazole	− 12.336
		Voriconazole	− 8.556
*A. niger*	D (Q228R/R501Q)	Itraconazole	− 12.103
		Voriconazole	− 8.661
*A. niger*	E (Q228R/I244F/D253Y/E254	Itraconazole	− 13.479
	D/T267Y/Y268H/E278Q)	Voriconazole	− 8.891
*A. niger*	F (wild-type)	Itraconazole	− 12.017
		Voriconazole	− 8.055
*A. tubingensis*	G (V329I, L492M)	Itraconazole	− 12.154
		Voriconazole	− 8.032
*A. tubingensis*	H (T321A)	Itraconazole	− 11.561
		Voriconazole	− 8.117
*A. tubingensis*	I (T321A, V377I)	Itraconazole	− 12.723
		Voriconazole	− 8.610
*A. tubingensis*	J (V329I)	Itraconazole	− 12.272
		Voriconazole	− 8.526
*A. tubingensis*	K (T321A, K477N)	Itraconazole	− 12.983
		Voriconazole	− 8.40
*A. tubingensis*	L (wild-type)	Itraconazole	− 10.992
		Voriconazole	− 8.230

The obtained homology-modeled protein of each isolate was aligned individually with wild-type Cyp51A modeled protein. The differences in protein backbone structures are quantitated with the root mean square deviation (RMSD) of the similarity between two superimposed atomic coordinates of modelled proteins. The RMSD scores demonstrated high similarity among models A to E when compared to the susceptible *A. niger* model (model F), with all models exhibiting a difference of less than 0.1 Å. In the case of *A. tubingensis*, the RMSD scores also demonstrated substantial similarity among models G to K compared to the susceptible *A. tubingensis* model (model L), all models displaying a difference of less than 0.24 Å (Fig. 3). Table 4 depicted the RMSD values obtained (because of mutations) of the aligned protein models of the mutated sequences with wild-type Cyp51A of *A. niger* and *A. tubingensis*, respectively.

**Table 4:** RMSD values of the aligned Cyp51A models of the mutated isolates with wild-type Cyp51A of *A. niger* and *A. tubingensis*.

Species	Model	Mutations	RMSD of modeled mutated Cyp51A with wild-type protein model (Å)
*A. niger*	A	Q228R/V383L	0.614
	B	Q228R/S346R	0.588
	C	Q228R	0.541
	D	Q228R/R501Q	0.530
	E	Q228R/I244F/D253Y/E254/D/T267Y/Y268H/E278Q	0.571
*A. tubingensis*	G	V329I, L492M	0.703
	H	T321A	0.807
	I	V377I, T321A	0.783
	J	V329I	0.657
	K	K477N, T321A	0.891

### Gene expression analysis

**Figure 4:**
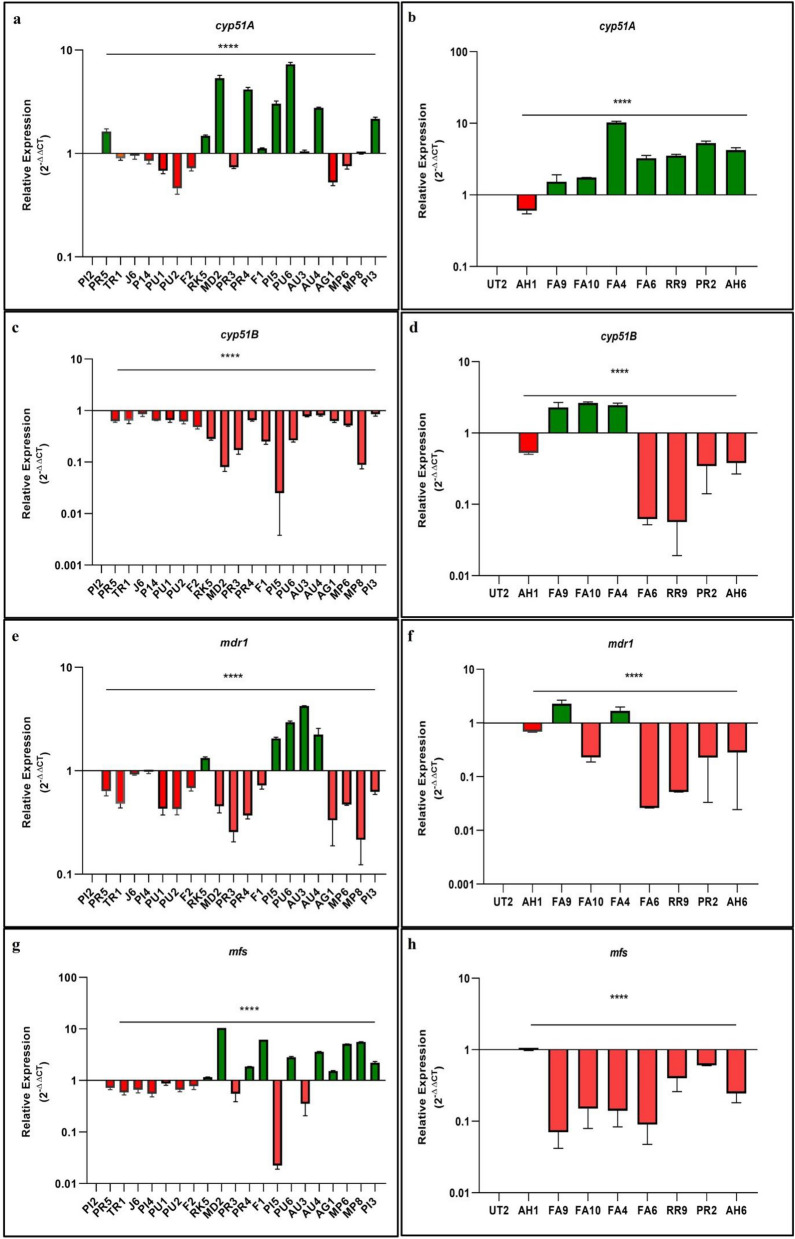
Relative expression of *cyp51A* ((**a**) and (**b**))*, cyp51B* ((**c**) and (**d**))*, mdr1* ((**e**) and (**f**)), and *mfs* ((**g**) and (**h**)) genes in azole-resistant environmental isolates of *A. niger* and *A. tubingensis*, respectively. In *A. niger* isolates PI2, PI4, PU1, PU2, F2, TR1, PR5, J6 were the azole-susceptible while isolates RK5, MD2, PI3, PR3, PR4, F1, PI5, PU6, AU3, AU4, AG1, MP6, MP8 were azole-resistant. In *A. tubingensis* UT2 and AH1 were the susceptible isolates while FA9, FA10, FA4, FA6, PR9, PR2, AH6 were resistant isolates. Azole-susceptible isolates PI2 and UT2 were the reference isolates for *A. niger* and *A. tubingensis*, respectively. Actin is used as a housekeeping gene to normalize transcription level. Green-colored bars represent the upregulation of the gene, and red-colored bars represent the downregulation of the gene. ****p < 0.0001.

Reverse transcription analysis from the RNA samples followed by qRT-PCR was performed to understand the expression modulation of transcripts encoding for *cyp51A*, *cyp51B, mdr1*, and *mfs* genes in the azole-resistant isolates of *Aspergillus* section *Nigri*. Figure 4 indicates the two-fold relative expression of crucial genes in *Aspergillus* section *Nigri* isolates compared to the susceptible isolates PI2 of *A. niger* and UT2 of *A. tubingensis.*

Results depicted a consistent upregulation (> 3 folds) of *cyp51A* gene in isolates exhibiting exclusive resistance to ITC (MD2, PU6, PI5, and PR4), with the exception of PR3 and F1 isolates. Additionally, a slight upregulation (1.5 folds) of this gene was observed in a ITC resistant isolate RK5. Upregulation was also noticed in two out of the three isolates (AU4 and PI3) that showed cross-resistance to ITC and POS. Notably, the *cyp51A* gene showed a 1.7 folds upregulation in a susceptible isolate (PR5). However, in isolates with cross-resistance to ITC and VRC or isolate resistant only to POS, no upregulation was observed. In case of *A. tubingensis* isolates, the *cyp51A* gene consistently showed upregulation in all the isolates, except the susceptible isolate AH1. The expression analysis of the *cyp51B* gene revealed downregulation in all *A. niger* isolates including susceptible and resistant isolates while the gene was > 2 folds upregulated in three azole-resistant (FA4, FA9 and FA10) *A. tubingensis* isolates*.* Relative expression of gene *mdr1* showed over 2 folds increased expression in two ITC resistant isolates (PU6, and PI5) and one ITC resistant isolate (RK5) with a 1.5 folds upregulation. Additionally, there was a 4.19 folds upregulation in an isolate displaying cross-resistance to ITC and VRC (AU3), and 2 folds upregulation in an isolate with cross-resistance to ITC and POS (AU4). In the case of *A. tubingensis,* the gene was 2 folds upregulated only in two ITC resistant isolates (FA4 and FA9).Among the 13 *A. niger* isolates resistant to azoles, the expression of the *mfs* gene was found to be upregulated in five isolates resistant to ITC, three isolates displaying cross-resistance to ITC and POS, in one isolate resistant to POS, and one isolate demonstrating cross-resistance to ITC and VRC. Notably, the *mfs* gene exhibited high expression, with a 10 folds increase in isolate MD2, 6.19 folds in isolate F1, and a 5 folds upregulation in MP6 and MP8. Whereas, the *mfs* gene was downregulated in all *A. tubingensis* isolates*.*

It’s noteworthy that overexpression of both the *mdr1* and *mfs* gene was not observed in any of the susceptible *Aspergillus* section *Nigri* isolates. The expression data were normalized by housekeeping gene actin.

## Discussion

The members of *Aspergillus* section *Nigri* causes several human diseases such as keratitis and invasive aspergillosis^30,31^. However, the in vivo efficacy of antifungal therapy against the clinical isolates of black aspergilli is undetermined, and *in-vitro* data is limited. Several authors from different countries have highlighted the prevalence of azole resistance in *A. fumigatus*^4,32,33^. However, limited number of studies has reported *in-vitro* antifungal susceptibilities and resistance mechanisms in the black *Aspergillus* spp.

In our study, we isolated 161 black aspergilli from soil samples across diverse regions in India. Azole susceptibility assay identified 20 azole resistant isolates. Notably, the number of the resistant isolates was prominent in states known for extensive agricultural activities, including Haryana (7/46), Bihar (3/23), Punjab (4/12), Madhya Pradesh (2/10), Uttar Pradesh (1/10), and Assam (3/6). We hypothesized that this prevalence might be linked to the use of azole fungicides in these regions. This hypothesis aligns with similar observations in studies involving azole-resistant *A. fumigatus* isolates found in soils exposed to fungicides^34,35^. The absence of resistant isolates in other states like West Bengal despite being a major agriculture state may be due to the limited number of samples collected. Further studies with more extensive sampling are warranted. Azole susceptibility testing of these 161 isolates demonstrated that ITC resistance (19/161) was more common suggesting that ITC resistance may be more obvious among the species of black aspergilli. Similar result was observed in previous study conducted by Howard et al.^15^. In contrast, POS was the most effective azole against *Aspergillus* section *Nigri* isolates. Several authors have reported similar results^17,32,36^. Interestingly, cross-resistance for the three tested azole drugs were not obtained in our study.

Further, the molecular identification of these 30 isolates revealed that 21 as *A. niger* and 9 isolates as *A. tubingensis*. Other *Aspergillus* section *Nigri* isolates, such as *A. welwitschiae* and *A. brasiliensis*, were not identified in our dataset, despite being present in previous studies^21,37^. This discrepancy may be attributed to variations in geographical distribution.

We also tried to correlate the relation between azole drug resistance and mutations in the *cyp51A* gene of *Aspergillus* section *Nigri* isolates in this study.

Sequence analysis of the *cyp51A* gene revealed that amino acid alterations, including Q228R, S346R, V329I, and T321A were present in susceptible and resistant *Aspergillus* section *Nigri* isolates. Several new mutations (R501Q, I244F, E254D, D253Y, T267Y, Y268H, E278Q, and L303M) have been observed in ITC resistant isolates only. However, it remains unclear whether these mutations are responsible for azole resistance in these isolates or not, Hence, Cyp51A protein modeling was conducted to further examine the role of these mutations in azole resistance. Molecular docking results revealed docking scores for the drugs, and the most negative docking score was obtained with model E (Mutations, Q228R/I244F/D253Y/E254/D/T267Y/Y268H/E278Q) for ITC. The docking score was almost similar in all the models for VRC. Docking results suggested no significant change in the binding site of the Cyp51A protein due to amino acid substitutions. The modeling results and similarity between the protein structures (RMSD score) suggested that *cyp51A* mutations in these isolates do not cause a marked change in the overall protein structure and do not directly interfere with their binding to azole drugs to confer resistance. Despite the *in-silico* findings, it is crucial to acknowledge the limitations of computational models. To validate the real impact of these amino acid substitutions, further *in-vitro* experiments employing the CRISPR/Cas9 system are essential. The approach has previously been employed to study the *cyp51A* gene in *Aspergillus* species^38,39^.

This study also demonstrated that mutations Q228R, S346R, and T321A were not specific to a particular azole drug. The presence of these mutations were observed in different isolates displaying varied resistance viz*,* ITC or POS, as well as in isolates exhibiting cross-resistance to ITC and VRC or ITC and POS. This diversity in mutation patterns suggested a lack of uniformity in the correlation between specific mutations and resistance profiles.

Similar to the study conducted by Howard et al.^15^, which also proposed that mutations in the *cyp51A* gene may not be crucial for azole resistance in section Nigri.

Azole resistance is mainly associated with acquiring genetic mutations or overexpression of the *cyp51A* gene and genes associated with efflux pump^40^. The overexpression of the *cyp51A* gene plays a crucial role in azole resistance in *A. fumigatus*; therefore, the expression of the *cyp51A* gene was also investigated in all 30 *Aspergillus* section *Nigri* isolates. Our findings revealed that the expression of *cyp51A* gene was significantly (*p* value ≤ 0.05) upregulated in seven azole-resistant *A. niger* isolates when compared to the susceptible isolate PI2. Similarly, the gene exhibited overexpression in all the resistant *A. tubingensis* isolates in comparison to the susceptible isolate UT2.

Surprisingly, even a susceptible *A. niger* isolate (PR5) carrying the Q228R mutation in the *cyp51A* displayed significant overexpression of this gene. This observation implies that the overexpression of the *cyp51A* gene may not always result in azole resistance in this fungal species. These results were consistent with the observations reported in previous studies^21,37^. Furthermore, we found that the *cyp51B* gene was downregulated in both the resistant and susceptible *A. niger* isolates in comparison to susceptible isolate PI2. Conversely, in three ITC-resistant isolates (FA4, FA9, and FA10), characterized by the presence of T321A in combination with V377I or K477N mutations, where the *cyp51B* gene exhibited upregulation compared to susceptible UT2 isolate. The baseline expression of *cyp51A* was more than that of *cyp51B.* However, a recent study on ergosterol quantification has revealed that both enzymes have a comparable impact on the total ergosterol content within the *Aspergillus* section *Nigri* cell^41^.

In *Candida albicans* and *Candida glabrata*, overexpression of efflux pumps, ATP-binding cassette transporters, and transporters of the major facilitator superfamily has been extensively studied^42^. Other studies have highlighted the significance of efflux pump genes overexpression in azole resistant *A. fumigatus* as well^28,29^. However, to the best of our knowledge, the expression of efflux pump genes in *Aspergillus* section *Nigri* isolates in India has not been investigated. In a recent study overexpression of these genes in *Aspergillus* section *Nigri* isolates has been reported, suggesting a possible drug resistance mechanism^26^. Therefore, we analyzed the expression of efflux pumps genes. The relative expression of the MDR efflux pump gene *mdr1* was upregulated in five *A. niger* resistant isolates (AU3, AU4, PU6, RK5, and PI5) and two *A. tubingensis* resistant isolates (FA4 and FA9). The overexpression of *mdr1* gene was observed along with the overexpression of *cyp51A* and *mfs* gene in 3 isolates (AU4, PU6, and RK5) carrying Q228R mutation alone or in combination with S346R. 2 folds upregulation of the gene was also observed in PI5 isolate carrying mutation R501Q in combination with Q228R. Conversely in isolate AU3, carrying similar mutations (Q228R, S346R) showed 4 folds overexpression only for *mdr1* gene. Additionally, two *A. tubingensis* resistant isolates displayed elevated expression of the *mdr1* gene, along with overexpression of *cyp51A* and *cyp51B*. The diverse gene expression pattern even among isolate with similar mutation highlights the complex azole resistance mechanisms in these fungal isolates.

The *mfs* gene, was upregulated in 10 out of 13 resistant *A. niger* isolates, and the gene was downregulated in all *A. tubingensis* isolates*.* Remarkably, among these three *A. niger* isolates (AG1, MP6, and MP8) displaying diverse susceptibility patterns, a common mutation profile of Q228R in combination with S346R was associated solely with the upregulation of the *mfs* gene in these isolates. The *mfs* gene exhibited high expression, with a 10 folds increase in isolate MD2, 6.19 folds in isolate F1, and a 5 folds upregulation in MP6 and MP8.

However, it’s important to note that efflux pumps genes *mdr1* and *mfs* was not upregulated in all the susceptible *Aspergillus* section *Nigri* isolates. These findings suggest a possible association between the overexpression of these genes and drug resistance in *Aspergillus* section *Nigri* isolates. Previous studies have also investigated the overexpression of the *mdr1* gene in azole-resistant *Aspergillus flavus* isolates lacking mutations in the *cyp51A* region^43^. Additionally, reports in *A. fumigatus* have indicated the upregulation of the *mfs* gene without *cyp51A* mutation^44^. Furthermore, the overexpression of *mdr1* and *mfs* genes has also been reported in other studies in *A. flavus*^45,46^.

Although we identified mutations in the *cyp51A* gene of *Aspergillus* section *Nigri* isolates but our analysis using Cyp51A homology modeling did not show significant changes in protein structure due to these mutations. Therefore, we analyzed the role of efflux pumps in conferring resistance and found that 92% of *A. niger* resistant isolates exhibited overexpression of efflux pumps genes either *mdr1* or *mfs.* The overexpression of these genes may cause azole resistance in *Aspergillus* section *Nigri* isolates. However, in the context of *A. tubingensis* isolates, we noted overexpression of the efflux pump gene (*mdr1*) in only two of the isolates. Interestingly, one of the *A. niger* isolate (PR3) exhibited resistance to the drug ITC. However, the gene expression data for selected genes (*cyp51A, cyp51B, mdr1, and mfs*) did not show significant overexpression in comparison to sensitive isolate. This observation suggests the presence of an alternative mechanisms contributing to azole resistance. The potential mechanisms may involve the upregulation of ABC transporter genes such as *cdr1B* and *mdr4,* which could result in reduced drug concentrations with in these fungal isolates. To comprehensively understand the reasons behind this susceptibility profile and to explore potential resistance mechanisms further experiments will be required.

The excessive use of triazoles in agriculture leads to their accumulation in the environment leading to the development of azole resistance. These resistant strains may infect the immunocompromised individuals and are subsequently detected in clinical settings. Our previous research, as well as other studies, have reported instances of azole resistance in *A. fumigatus* in the environment due to the use of azole fungicides^33,47^. In the present study, the identification of azole-resistant *Aspergillus* section *Nigri* isolates from the same geographical area and the overexpression of efflux pump genes in these isolates without exposure to azole drugs, has prompted us to hypothesize. We suggests that the emergence of resistance in *Aspergillus* section *Nigri* might be linked to the environmental application of azole fungicides, potentially triggering a stress response leading to upregulation of efflux pump genes.

Therefore, large-scale epidemiological studies are required to monitor the resistant fungal strains in the environment. Further, the antifungal susceptibility profiles of environmental or clinical fungal isolates should be accurately monitored.

## Conclusion

The identified 15 amino acid substitutions in the *cyp51A* gene of *Aspergillus* section *Nigri* isolates in our study does not completely correlate with resistance data, which suggests, other genes such as *mdr/ mfs* needs to be investigated for mutational analysis. Advanced techniques such as CRISPR/Cas9 could be applied to study the genetic changes. The results in the study suggests that overexpression of *mdr1* and *mfs* genes could potentially play a role in drug resistance in *Aspergillus* section *Nigri.* Studies on expression of the *cyp51A, cyp51B*, and multidrug efflux transporter at transcript and protein levels will help identify other genes and proteins involved in resistance. Further, genome-wide profiling may provide a better understanding of the mechanisms of azole resistance in *Aspergillus* section *Nigri* isolates.

## Methods

### Environmental sampling and isolation of black *Aspergilli*

A total of 174 agricultural soil samples were collected from 19 states across India including Assam (n = 12), Bihar (n = 21), Chhattisgarh (n = 2), Delhi (n = 12), Haryana (n = 23), Himachal Pradesh (n = 3), Jammu and Kashmir (n = 1), Karnataka (n = 4), Maharashtra (n = 3), Manipur (n = 3), Madhya Pradesh (n = 10), Orissa (n = 4), Punjab (n = 12), Rajasthan (n = 9), Sikkim (n = 1), Tripura (n = 1), Uttaranchal (n = 8), Uttar Pradesh (n = 31), and West Bengal (n = 14).

All samples were processed and inoculated on potato dextrose agar (PDA) plates as previously described by Sen et al.^48^. The plates were incubated at 28 ± 2 °C for 5 days. All black aspergilli isolates grown on PDA plates were identified based on their macroscopic and microscopic morphologies^48^. The isolates were further sub-cultured on PDA and stored at 4 °C till further use.

### Antifungal susceptibility testing

The spores (conidia) of all the black aspergilli isolates were harvested in sterile phosphate buffered saline (1 × PBS) supplemented with 0.05% Tween 20; suspension was then adjusted to 1 × 10^4^ conidia/mL in potato dextrose broth. *In-vitro* susceptibility testing of black aspergilli isolates was performed to determine the MICs of ITC, VRC and POS using CLSI M38- A2 broth microdilution method^49^ in a 96-well flat bottom polystyrene plate (Tarsons, India). The experiment was performed in triplicate for each isolate. The azole drug stocks were prepared in dimethyl sulfoxide. Two-fold dilutions were prepared in a 96-well microplate to obtain final concentrations ranging from 64–0.125 µg/mL for ITC, VRC, and POS. Conidial suspension (100 µL) was added to each well except negative control. The plates were incubated statically for 4 days at 28 ± 2 °C. The proposed ECVs for ITC, VRC, and POS were 2, 2 and 0.5 µg/mL, respectively^50,51^. These values were used to interpret the results. The MIC value of a drug was determined as the lowest concentration with no visible growth relative to the drug-free control.

### Genomic DNA extraction

To identify the azole resistant black *Aspergillus* isolates and analyse their mutations related to azole susceptibility, we used the cetyl trimethyl ammonium bromide (CTAB) method^52,53^ to extract genomic DNA from 20 resistant black aspergilli isolates. Additionally, to establish a comparative context, we have selected an extra set of 10 azole susceptible black *Aspergillus* isolates from the same geographical region as the resistant isolates.

### Molecular identification of black *Aspergillus* isolates

All the 30 isolates (20 resistant and 10 susceptible) were identified by the amplification and sequencing of full-length 18S ITS region, partial sequencing of *β*-tubulin genes using the ITS1 and ITS4^54^ and Tub5 (5' TGACCCAGCAGATGTT 3') and Tub6 (5' GTTGTTGGGAATCCACTC 3')^55^ primers, respectively as previously described^47^.

### Sequencing of *cyp51A* gene

Different primer sets were used for amplification of the *cyp51A* gene of twenty resistant and ten susceptible isolates (Table S1)^21^. Nucleotide sequencing was performed via Sanger’s sequencing using ABI 370 XL (Applied Biosystems). The sequence of the products was compared to the *A. niger* (Accession no. NT_166526) and *A. tubingensis* (Accession no. JF450924.1) *cyp51A* wild-type sequence using the NCBI alignment service, Align Sequence Nucleotide BLAST (https://blast.ncbi.nlm.nih.gov/Blast.cgi) and Clustal Omega tool (https://www.ebi.ac.uk/Tools/msa/clustalo/).

### Homology modeling

Cyp51A protein structure of *A. niger* and *A. tubingensis* were not available on RCSB-PDB hence; FASTA sequence of *A. niger* (XP_001394224.1) and *A. tubingensis* (AEK81606.1) were retrieved from NCBI to construct the homology models of the wild type strains. Amino acid substitutions were incorporated in the reference protein sequences of *A. niger* and *A. tubingensis* to perform homology modeling. The X-ray crystal structures of protein 4UYM and 5FRB were used as reference for the model generation of *A. niger* and *A*. *tubingensis* based on the sequence similarity. The Schrödinger Maestro multiple sequence viewer (MSV) tool^56^ was used to construct a structure-based alignment of the mutated templates. The template structure for alignment was identified through searching the wild type sequences using the BLAST tool incorporated in Schrodinger Maestro multiple sequence viewer. The homology models were built with Prime in Schrödinger Suite (Schrödinger, LLC, New York, NY). Models were refined using minimization and loop refining tool of prime. The results of homology modeling were further validated using Ramachandran plot. Modeled structures were aligned and root mean square deviation (RMSD) was calculated using Maestro Schrödinger.

### Molecular docking

The 3D structures of VRC (PubChem ID- 71,616) and ITC (PubChem ID- 55283) were retrieved from the PubChem (https://pubchem.ncbi.nlm.nih.gov/). The 3D structures of these drugs were prepared using Ligprep in Schrödinger Suite and possible states were generated at pH 7.0 ± 2.0 (LigPrep 2022). The homology models were prepared for docking using one-step protein preparation workflow in Schrödinger Maestro (Maestro 2022) by adding and refining missing hydrogen atoms. Molecular docking calculations were completed using Glide in Schrodinger docking suits (Glide 2021)^57^. Modeled proteins were prepared by restrained minimization using force field OPLS3e. Grids centers were determined from ligands of reference proteins. Receptor grid maps representing the shape and chemical properties of the binding site were generated using Schrödinger Glide. The binding site of modeled proteins was also confirmed by the Sitemap predicting possible binding pockets. The grid sites were created using Glide receptor grid generator with docking length of 20 Å. Docking was performed using the Schrödinger Virtual Screening Workflow tool using Glide Standard Precision (SP) with the OPLS3e force field^58^. One pose per ligand was kept for post-docking full force field minimization (optimization of ligand pose geometry followed by recalculation of interaction strength between ligand–protein using the scaled Coulomb-van der Waals term and the Glide score). Docking scores are reported in kcal/mol, the more negative the number, the better binding.

### Quantification of gene expression by qRT-PCR

To assess the expression level of the *cyp51A*, *cyp51B, mdr1,* and *mfs* genes in thirty *Aspergillus* section *Nigri* isolates, quantitative real time reverse transcription PCR (qRT-PCR) was used. Further, to carry out qRT-PCR, RNA was extracted from the harvested mycelia using TRIzol reagent (Thermo Fisher)^59,60^. This experiment was carried out in the absence of azole drug exposure. The extracted RNA was then reverse transcribed into first-stand cDNA using the Hi-cDNA synthesis kit (HiMedia, India) by following the manufacturer’s recommendations. Real-time qPCR was performed using an ABI QuantStudio 3 (Applied Biosystems, Streetsville, Canada) as previously described in a study by Gupta et al.^60^. The gene expression was estimated using the 2^−ΔΔCt^ method, with actin as the reference gene^21^.

Accession numbers XM_001398369.2 and XM_035497978.1 were used for the primer designing of *mdr1* and *mfs* gene, respectively. The gene specific primers were designed using the Primer 3 software (http://primer3.ut.ee/). The primer sets used in this study are listed in Table S1.

### Statistical analysis

One-way ANOVA test was used to compare relative gene expression levels. The experiment was conducted in biological and technical triplicate. The *p* value of ≤ 0.05 was considered significant. Statistical analysis was also performed using GraphPad Prism v8.0.2.263.

### Supplementary Information

Supplementary Information.

## Data Availability

Data are presented within the manuscript and in the Supplementary Information.
