# Supplementary material for: cyp51A mutations, protein modeling, and efflux pump gene expression reveals multifactorial complexity towards understanding Aspergillus section Nigri azole resistance mechanism
Source: Sci Rep. 2024 Mar 14;14:6156. doi: 10.1038/s41598-024-55237-9 (PMC10940716; doi:10.1038/s41598-024-55237-9)
Supplement: Supplementary file 1 — Supplementary Information. [file 41598_2024_55237_MOESM1_ESM.docx]

***cyp51A* Mutations, Protein Modeling, and Efflux Pump Gene Expression Reveals Multifactorial complexity towards understanding *Aspergillus* Section *Nigri* Azole Resistance mechanism**

**Pooja Sen^1^, Mukund Vijay^1^, Himanshu Kamboj^1^, Lovely Gupta^1^, Jata Shankar^2^ and Pooja Vijayaraghavan^1^***

^1^Amity Institute of Biotechnology, Amity University Uttar Pradesh, Sector-125, NOIDA, Uttar Pradesh, India

^2^Department of Biotechnology and Bioinformatics, Jaypee University of Information Technology, Solan, India

***Corresponding author: Pooja Vijayaraghavan, Ph.D.,** Amity Institute of Biotechnology, Amity University Uttar Pradesh, Sector-125, NOIDA, Uttar Pradesh, India

Email: [vrpooja@amity.edu](mailto:vrpooja@amity.edu)

**Supplementary Table S1** Primers used for qRT-PCR and sequencing in *Aspergillus* section *Nigri.*

| **S.No.** | **Gene name** | **Primer** | **Primer’s Name** | **Primer sequence (5ʹ-3ʹ)** | **Use** | **References** |
| --- | --- | --- | --- | --- | --- | --- |
| 1. | *actin* | Forward*^a^* | Act-F2 | ACCCTCAGATACCCCATTGA | qRT-PCR | [18] |
|  |  | Reverse*^a^* | Act-R2 | CTGGGTCATCTTCTCACGG | qRT-PCR | [18] |
| 2. | *cyp51A* | Forward*^a^* | cyp51A-F1 | GAACCCAGACGAGGAGAAG | qRT-PCR/seq | [18] |
|  |  | Reverse*^b^* | cyp51A-R1 | TCGCAGCATGATCCAAGAAC | qRT-PCR/seq | [18] |
|  |  | Reverse*^c^* | cyp51A-R2 | CGCAACATAATCCAAGAGCTA | qRT-PCR/seq | [18] |
|  |  | Reverse*^b^* | An-R3 | GCTCCCAACCCACTATAGC | seq | [18] |
|  |  | Forward*^b^* | An-F2 | CGATAGTCTTAAATGTCACGC | seq | [18] |
|  |  | Forward*^c^* | Aspbt-F2 | TGCTCGTTGCGATAGTCTTG | seq | [18] |
|  |  | Reverse*^c^* | Asptu-R1 | TTAGTTCAAGGACCCCTTGGA | seq | [18] |
| 3. | *cyp51B* | Forward*^a^* | cyp51B-F1 | GTTTCCATTCATAGGTAGCAC | qRT-PCR | [18] |
|  |  | Reverse*^a^* | cyp51B-R1 | ACTTCTTCAGCACAGACATCA | qRT-PCR | [18] |
| 4. | *mdr1* | Forward*^a^* | mdr1-F1 | ACGGAGACTAAGCATCTTTC | qRT-PCR | - |
|  |  | Reverse*^a^* | mdr1-R1 | CAAGCATGTAGAAACGGTAG | qRT-PCR | - |
| 5. | *mfs* | Forward*^a^* | mfs-F1 | AGTAATGTGGGTGTGATGAG | qRT-PCR | - |
|  |  | Reverse*^a^* | mfs-R1 | GTGCTCAAGTGTTCAAGGAG | qRT-PCR | - |

*^a^*Primers used in this study for both the isolates (*A. niger* and *A tubingensis).*

*^b^*Primers used in this study for *A. niger* isolates only.

*^c^* Primers used in this study for *A. tubingensis* isolates only.

**Supplementary Figure S1** Sequence alignment of the Cyp51A proteins of *Aspergillus niger* isolates with *A. niger* (NT_166526) sequence. Identical residues are marked in blue and green shades, and amino acid substitutions detected in the Cyp51A sequence of the isolates are highlighted in red. **
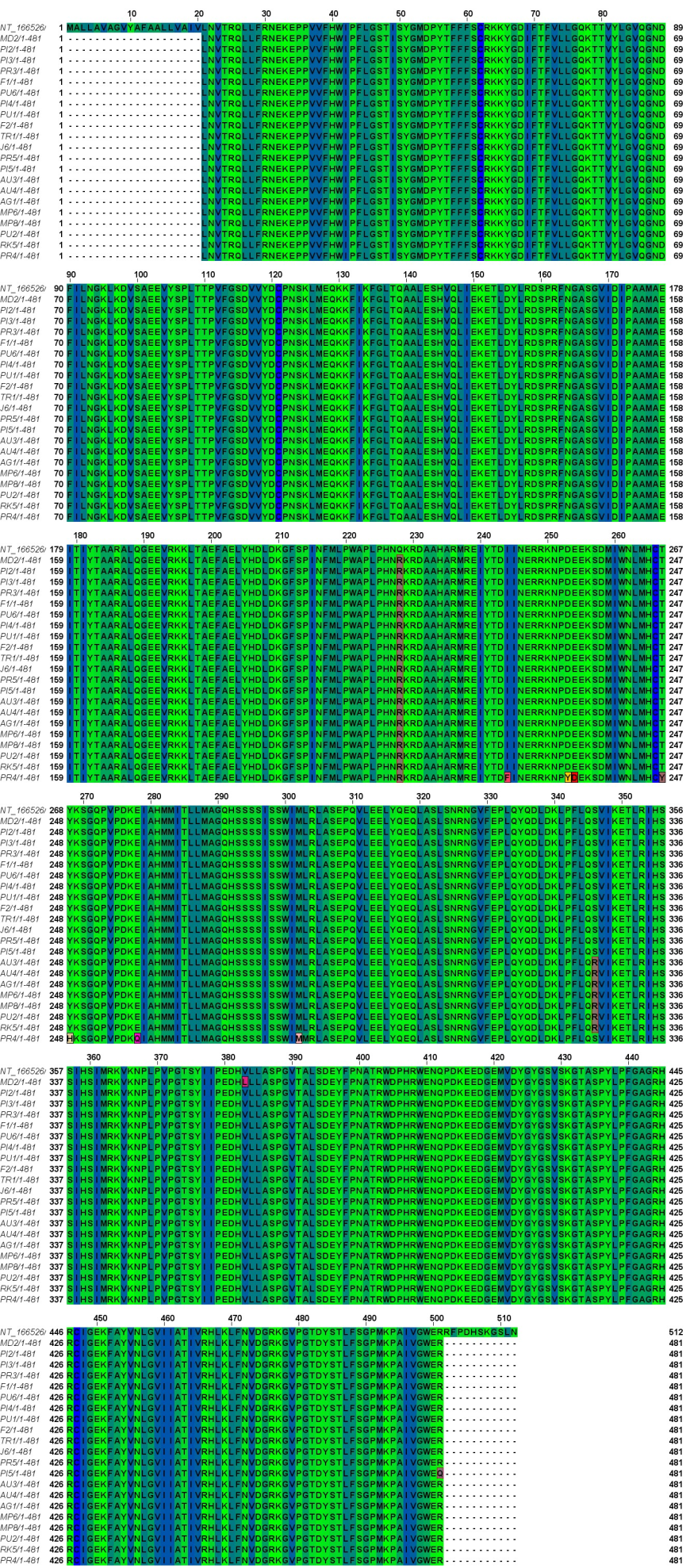
**


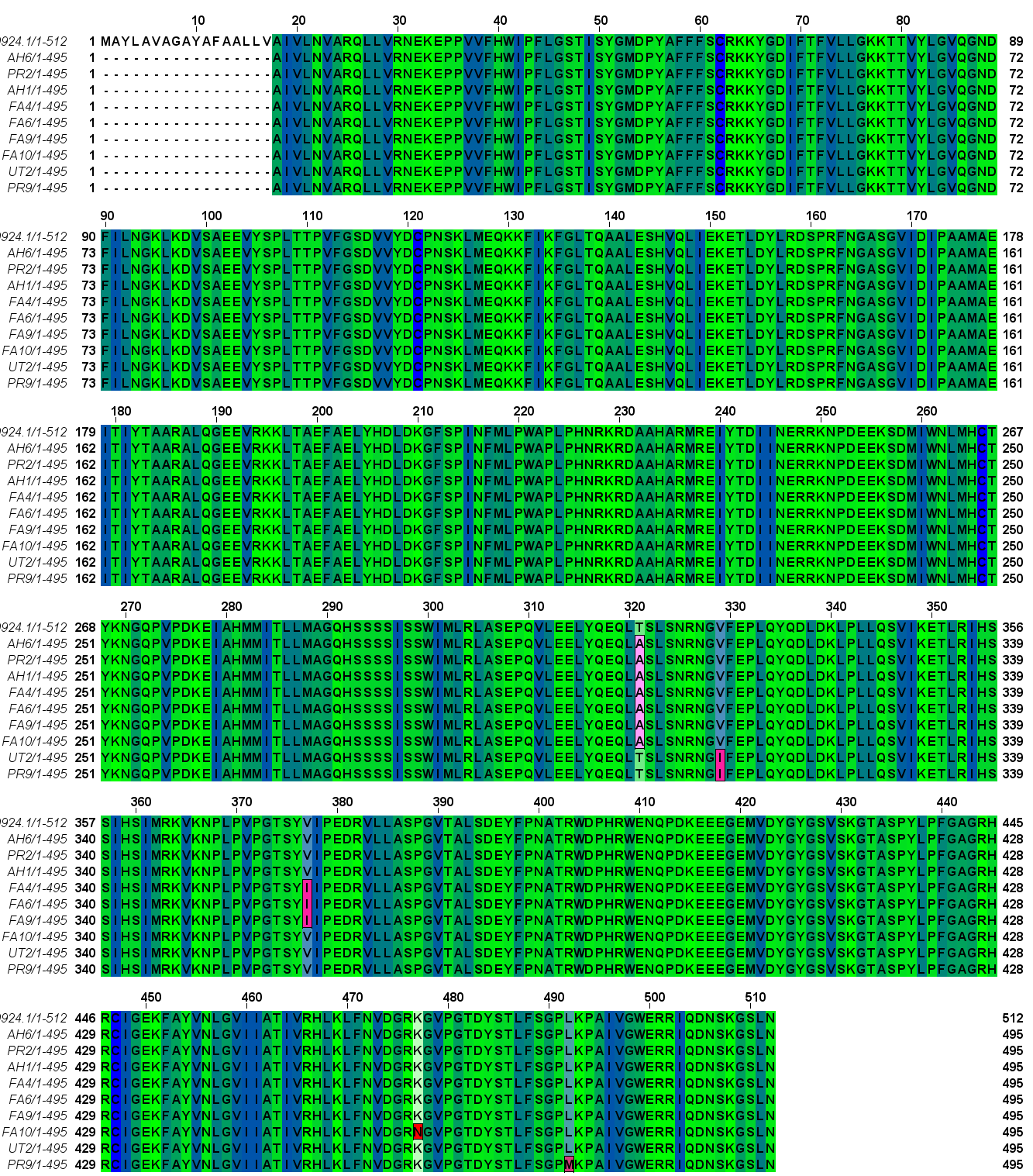
**Supplementary Figure S2** Alignment of *A. tubingensis* (JFA450924.1) Cyp51A proteins sequence with the sequences from *A. tubingensis* isolates. Identical residues are marked in blue and green shades, and amino acid substitutions detected in the Cyp51A sequence of the isolates are highlighted in red.
